# Dual-species biofilms of *Streptococcus mutans* and *Candida albicans* exhibit more biomass and are mutually beneficial compared with single-species biofilms

**DOI:** 10.1080/20002297.2019.1581520

**Published:** 2019-03-20

**Authors:** Carmélia Isabel Vitorino Lobo, Talita Baptista Rinaldi, Chiara Mikaella Somogyi Christiano, Luana De Sales Leite, Paula Aboud Barbugli, Marlise Inêz Klein

**Affiliations:** Department of Dental Materials and Prosthodontics, São Paulo State University (Unesp), School of Dentistry, Araraquara, Brazil

**Keywords:** Biofilm, *Streptococcus mutans*, *Candida albicans*, stress tolerance, gene expression, 3D architecture

## Abstract

**Background**: *Streptococcus mutans* (*Sm*) and *Candida albicans* (*Ca*) are found in biofilms of early childhood caries. **Objective:** To characterize* in vitro* dual- and single-species biofilms of *Sm* and *Ca* formed on saliva-coated hydroxyapatite discs in the presence of sucrose. **Design: **Evaluation of biofilms included biochemical [biomass, proteins, matrix’s water-soluble (WSP) and alkali-soluble (ASP) polysaccharides, microbiological, 3D structure, gene expression, and stress tolerance analyses. **Results: **Biomass and proteins were higher for dual-species and lower for *Ca* (p = 0.001). Comparison of *Sm* single- and dual-species biofilms revealed no significant difference in *Sm* numbers or quantity of WSP (p > 0.05). Dual-species biofilms contained a higher population of *Ca* (p < 0.001). The quantity of ASP was higher in dual-species biofilms (*vs Ca* single-species biofilms; p = 0.002). The 3D structure showed larger microcolonies and distinct distribution of *Sm-*derived exopolysaccharides in dual-species biofilms. Compared with dual-species biofilms, expression of *gtfB* (ASP) and *nox1* (oxidative stress) was higher for single-species of *Sm* whilst expression of BGL2 (matrix), PHR1 (matrix, acid tolerance) and SOD1 (oxidative stress) was higher in single-species of *Ca*. There was no difference for acid tolerance genes (*Sm atpD* and *Ca* PHR2), which was confirmed by acid tolerance challenge. Dual-species biofilms were more tolerant to oxidative and antimicrobial stresses (p < 0.05). **Conclusions: **Dual-species biofilms present greater 3D complexity, thereby, making them more resistant to stress conditions.

## Introduction

Dental caries is a biofilm-diet-dependent disease that causes tooth demineralization by acids produced by microorganisms via the metabolism of dietary carbohydrates [1]. Early childhood caries or ECC is an aggressive form of the disease with high prevalence in both developed and developing countries, is challenging to treat and highly costly. Children affected with ECC have a diet rich in carbohydrates, such as sucrose, which promotes the formation of microbial communities that are predominated by cariogenic/aciduric microorganisms and consequent accumulation of virulent biofilms culminating with rapid destruction of dental tissue [2].

Biofilms are highly dynamic, three-dimensional (3D) structured and organized communities of microbial cells; the cells are covered and immersed by an extracellular matrix of polymeric substances such as exopolysaccharides [3]. *Streptococcus mutans* is still one of the main microorganisms associated with caries because it orchestrates the construction of the cariogenic biofilms by controlling the assembly of a matrix rich in insoluble exopolysaccharides, mainly α1,3-glucans [1,4]. Furthermore, it produces acids and survives in the acidic environment [5]. Insoluble exopolysaccharides prevent the saliva neutralization of acids produced by microorganisms [6]. In addition, *Candida albicans* has also been found in high numbers in cariogenic biofilms (mainly in ECC) [7–10]. This fungus also produces acids and survives in an acidic environment, synthetize proteinases that degrade collagen (among other exoenzymes [11,12]), which may contribute to the biofilm’s cariogenicity. Still, *C. albicans* is not recognized as a true pathogen for dental caries; however, it may act as a secondary agent in caries lesions process (especially in dentin) [13].

A bacterium-fungal association may be antagonistic or cooperative. It is cooperative when microorganisms provide substrates and/or metabolites or stimulate one another. For example, *C. albicans* does not efficiently metabolize sucrose and is favored by degraded products of sucrose by *S. mutans* (glucose and fructose). In contrast, the presence of *C. albicans* in the biofilm alters the physical environment, favoring the increase of exopolysaccharides and consequently, accumulation and formation of microcolonies by *S. mutans* [14,15]. In the presence of sucrose, *S. mutans* produces exoenzymes glucosyltransferases (Gtfs), which bind to the tooth enamel and *C. albicans* leading to exopolysaccharides accumulation on both surfaces [15,16]. Thus, *C. albicans* and *S. mutans* develop a symbiotic interaction mediated by Gtfs, particularly GtfB [14,16]. *In vitro* biofilms formed by *S. mutans* and *C. albicans* differ greatly from those formed by *S. mutans* only, where the presence of *C. albicans* induces the expression of *S. mutans gtfB* and genes to cope with environmental stress (e.g. *atpD* linked to acid stress tolerance) [14]. Moreover, compared with single-species infection, the co-infection of animals, with both microorganisms, leads to a higher number of carious lesions which are also more severe [14,15].

In addition, *C. albicans* produces β-glucans that are part of the cell wall and are also found in the matrix of the biofilm. Of note is that antifungal resistance is associated with the presence of β-1,3 and β-1,6 glucans [17–19]. The expression of *C. albicans* genes is also important for biofilm formation and cells tolerance to several environmental stresses. For example, the gene expression of the glucan transferase BGL2 (synthesis of β-glucans) [19] and the glycosidase PHR1 (important in cell wall structure and virulence of infections related to low pH) [20] are associated with the construction of the extracellular matrix by *C. albicans* [19]. In contrast, the glycosidases PHR2 plays an essential role in cell wall structure while SOD1 (copper and zinc cytosolic superoxide dismutase) is involved in protection against oxidative stress and is critical for virulence [20–22]. Both PHR1 and PHR2 are required for proper cross-linking of β-1,3 and β-1,6 glucans [23]. Nevertheless, the pattern of expression of these genes in dual-species biofilms of *S. mutans* and *C. albicans* is not well understood.

Furthermore, it has been demonstrated that strategies used to control *S. mutans* single-species biofilms [24] had little effect on dual-species biofilms of *S. mutans* and *C. albicans* [25]. In addition, exopolysaccharides produced by *S. mutans* enhanced antifungal drug tolerance in dual-species biofilm [26]. Therefore, there is a need for further understanding of *S. mutans* and *C. albicans* cariogenic biofilms to develop new effective therapies. Thus, the present study characterized *in vitro* single- and dual-species biofilms of *S. mutans* and *C. albicans* via microbial population (viable counts of microbial cells), biomass, exopolysaccharides in the matrix, structural organization, gene expression and response to environmental stresses (acidic, oxidative, and antimicrobial).

## Methods

### Biofilm formation in vitro

The strains *S. mutans* UA159 and *C. albicans* SC5314 were cultured on blood agar plates for 48 h (37ºC, 5% CO_2_ – LABOVEN, model L212). Five colonies of each strain were transferred separately to liquid culture medium (tryptone with yeast extract – TYE, pH 7.0 Difco) containing 1% of glucose (w/v). After incubation (37ºC, 5% CO_2_, 16 h), these starter cultures were diluted 1:20 in the same medium and incubated until reaching the mid-log growth phase (OD _600 nm_ 0.710 ± 0.270 for *S. mutans*, and 0.970 ± 0.030 *C. albicans*) [25]. Next, the tubes containing each strain were centrifuged (4000 rpm, 20 min, 4ºC; Eppendorf, Centrifuge 5810R). The spent medium was discarded, the pellet resuspended with TYE supplemented with 1% sucrose (w/v), and the optical density was rechecked to confirm the number of cells. These cultures were diluted to obtain inocula to grow biofilms.

Hydroxyapatite (HA) discs with the total surface area of 2.7 ± 0.2 cm^2^ (Clarkson Chromatography Products, Inc., South Williamsport, PA), were used as the surface for biofilm formation, to mimic the dental enamel. Total area of discs was used for all analyses, except for the microscopy in which only a face of disc was selected. These discs were coated with saliva for acquired pellicle formation, following a previous protocol [27]. Stimulated whole saliva was obtained from three healthy volunteers, diluted 1:1 with adsorption buffer [AB buffer: 50 mM KCl, 1 mM KPO_4_, 1 mM CaCl_2_, 1 mM MgCl_2_, 0.1 mM PMSF (phenylmethylsulfonyl fluoride – Sigma), in dd-H_2_O, pH 6.5], centrifuged (4000 rpm, 20 min, 4ºC – Eppendorf, Centrifuge 5810R) and sterilized by filtration (0.22 *μ*m low protein binding polyethersulfone membrane filter) [27]. The study was approved by the Institutional Ethical Committee (CAAE: 55.823.016.7.0000.5416). The HA discs were positioned vertically in 24-well plates using wire apparatus to avoid the effect of microbial deposits due to gravity [27] and incubated (75 rpm, 1 h, 37ºC) for the formation of the salivary pellicle, and then dip-washed twice into AB buffer.

After formation of the salivary pellicle, the dual-species biofilm inoculum used was formed with UA159 (2x10^8^ colony forming units per milliliter – CFU/mL) and *C. albicans* SC5314 (2x10^6^ CFU/mL) [25]. Single-species biofilms of *S. mutans* (2x10^8^ CFU/mL) and *C. albicans* (2x10^6^ CFU/mL) were used as controls. The biofilms were inoculated in the liquid culture medium TYE containing 1% sucrose and incubated (37ºC, 5% CO_2_). The culture medium was changed when the biofilms reached the ages of 19 and 27 h by transferring biofilms to fresh culture medium TYE with 1% sucrose. Biofilms were evaluated at 19 h for the structural organization (confocal), 28 h (gene expression) and 43 h (quantification of viable microbial cell numbers, biomass, exopolysaccharides in the matrix, structural organization, and response to environmental stresses). The spent media was used to check the pH (pHmeter QX 1500 Plus).

### Laser scanning confocal fluorescence microscopy imaging of biofilms

Biofilms with 19 and 43 h of formation were analyzed via confocal microscopy. The stains and probes used are described in Table 1. The dual- and single-species biofilms of *S. mutans* UA159 and *C. albicans* SC5314 were formed as described above. For both 19 and 43 h-old biofilms, to label *S. mutans-*derived exopolysaccharides in the matrix, 13.4 μL of 1 mM Alexa Fluor 647 fluorophore-labeled dextrans were added to the culture medium (2.8 mL) at the beginning of biofilms growth [28,29].

**Table 1.** The probes and stains used in the confocal experiments.

Labeling Target	Labeling (stain or probe) (Excitation⁄ Emission nm):	Source	References
Exopolysaccharides produced by *S. mutans*	Alexa Fluor® 647-labeled dextran conjugate (647/668 nm)	Molecular Probes, Carlsbad, CA, USA (Cat. No. D22914)	[28]
*S. mutans*	UA159 strain that expresses a fluorescent green protein (GFP)	GFP lab construct	[14]
*C. albicans*	Concanavalin A (ConA) lectin conjugated with tetramethylrhodamine (555/580 nm)	Molecular Probes (Cat. No. C860)	
Both *S. mutans* and *C. albicans*	SYTO9 (485/498 nm)	Molecular Probes (Cat. No. S34854)	[14,28]
Polysaccharides produced by *C. albicans*	Primary monoclonal antibody to (1→3)-β-glucan	Biosupplies, Australia Pty Ltd., Victoria, Australia (Cat. No. 400–2)	[14,30,31]
	Primary monoclonal antibody to (1→3,1→4)-β-glucan	Biosupplies (Cat. No. 400–3)	
	Primary monoclonal antibody to (1→4)-β-mannan and galacto-(1→4)-β-mannan	Biosupplies (Cat. No. 400–4)	
Secondary antibody against the three primary antibodies	Goat Anti-Mouse IgG H&L (Alexa Fluor® 405) (401/421 nm)	Abcam (Cat. No. ab175660)	

For 19 h-old biofilms, the *S. mutans* UA159 strain used expresses a fluorescent green protein (GFP) and *C. albicans* cells were labeled with concanavalin A (ConA) lectin conjugated with the fluorophore tetramethylrhodamine (TRITC) (Table 1) [14]. Polysaccharides produced by *C. albicans* were stained using three commercially available mouse monoclonal IgG antibodies paired with a fluorescently labeled secondary antibody (Table 1).

The discs with 19 h-old biofilms were individually transferred to Petri dishes with coverslip bottom containing 0.89% NaCl solution. To each biofilm were added 150 *μ*L of primary antibody diluted 1:20 in 2% bovine serum albumin solution (BSA) and 0.1% Tripton-x100 (but no antibody for controls) and incubated for 18 h (4°C). After incubation, the biofilms were washed thoroughly with 0.89% NaCl solution and the blocking solution [phosphate buffered saline-PBS x1 (57 mM NaH_2_PO_4_, 50 mM Na_2_HPO_4_, and 200 mM NaCl; pH 7.33) + 3% of BSA] was added for 15 min (4ºC). Next, the biofilms were washed with 0.89% NaCl solution and the secondary antibody (1:500 dilution in 1x PBS) was added (but no antibody for controls), followed by incubation for 2 h (4°C). After this incubation, the biofilms were washed with 0.89% NaCl and incubated with 80 *μ*g/mL of ConA in NaCl 0.89% to stain the *C. albicans* cells for 30 min; followed by the last wash with 0.89% NaCl.

The images were acquired using a confocal fluorescence microscope (CARLS ZEISS LSM 800 with Airyscan with GaAsp detector, Germany), with a Plan Apochromat 63x/1.40 Oil DIC M27 objective. The detection parameters were (i) *S. mutans* single-species biofilms – 488 nm: 21% and 640 nm: 5.81% laser wavelengths; (ii) *C. albicans* single-species biofilms – 561 nm: 8.00% and 405 nm: 46.25% lasers wavelength; (iii) dual-biofilms – 405 nm (410–450): 55.00%, 488 nm (484–520): 24.00%, 561 nm (560–610): 9.00%, and 640 nm (656–700): 7.69% lasers wavelengths). The z-stack increments were 0.5 μm for single-species biofilms and 1.0 *μ*m for dual-biofilms. The images were analyzed using the ZEN Blue software (for 3D projections reconstruction). Detection wavelength allowed the observation and confirmation that the fluorescence emission of each laser was independent and ensured the absence of overlap of the labeled structures (cross-label).

In addition, 43 h-old dual- and single-species biofilms of *S. mutans* and *C. albicans* were performed for overall 3D architecture analysis of microbial microcolonies and exopolysaccharides produced by *S. mutans*. As described for 19 h-old biofilms, Alexa Fluor 647 fluorophore-labeled dextrans were added to the culture medium at the beginning of biofilms growth and during culture medium changes [28,29]. After 43 h of biofilm development, the microbial cells were evidenced with the SYTO9 fluorophore. The biofilms were transferred to wells containing 1.5 μL of SYTO9 and 2.8 mL of 0.89% NaCl solution and incubated at room temperature for 30 min. These biofilms were rinsed in 0.89% NaCl solution and transferred to Petri dishes with glass coverslip bottom containing 0.89% NaCl solution for image acquisition. The images were acquired using the same confocal fluorescence microscope (488 nm: 2.10% and 561 nm: 1.81% lasers wavelengths with EC Plan-Neofluar 20x/0.50 M27 objective), with 1024 × 1024 configuration in 1.5 μm increments. The images were analyzed using the ZEN Blue software. Two HA discs were used for each type of biofilm, and four experimental occasions were performed to obtain at least three images per disc (biofilm representative images).

### Gene expression

Expression of *S. mutans* (*gtfB, nox1, atpD*, and normalizer 16S rRNA) and *C. albicans* (BGL2, PHR1, PHR2, SOD1, and normalizers ACT1 and RPP2B) genes was measured by RT-qPCR (reverse transcript-quantitative polymerase chain reaction). This methodology included RNA isolation, cDNA synthesis, and gene expression analysis via qPCR. The single- and dual-species biofilms of *S. mutans* UA159 and *C. albicans* SC5314 were formed as described above.

#### RNA isolation

28 h-old dual- and single-species biofilms were removed from the culture medium, washed with saline solution (0.89% NaCl) and each biofilm disc was dispensed in a glass tube containing 2.5 mL of RNAlater (Ambion – Molecular Probes). The glass tubes containing discs with biofilms were sonicated in an ultrasonic bath (Kodortech, C-D-4820) for 10 min. Then, the remaining biofilm of each disc was removed by scraping with a spatula. The resulting biofilm suspension was then transferred to a 15 mL falcon tube. Next, 1 mL of RNAlater was used for washing of the glass tube and in the end was added to the first corresponding falcon tube 15 mL. The samples were stored in freezer −80°C until the extraction procedure.

RNA was isolated according to methodology optimized for biofilms [32], with some modifications (i.e. bead-beater time was increased from 40 s to 1 min). Briefly, the extraction of RNA was done using the phenol-chloroform separation method, and purification was via treatment with DNAse in column (Qiagen) and solution (TURBO DNAse; Ambion). DNAse was removed using the Rneasy MinElute clean-up kit (Qiagen). The integrity of purified RNA was determined by 1% agarose gel electrophoresis (Ultra-Pure ™ Invitrogen). Spectrophotometry was used to evaluate the amount (OD 260 nm) and purity (OD 260/280) of total RNA (Nano-spectrophotometer DS-11^+^, Denovix).

#### cDNA synthesis

The cDNA was synthesized using the iScript kit (BioRad). For cDNA synthesis, 0.5 μg of total RNA was mixed with four μL of the 5X iScript buffer, one μL of the reverse transcriptase enzyme, and molecular grade water to complete 20 μL (i.e. ^+^RT samples) [14]. RNA samples with all kit reagents except for reverse transcriptase were negative controls and used to determine DNA contamination (i.e. ^−^RT samples). The reactions were incubated using the CFX96 thermocycler (BioRad), following the cycle: 25ºC/5 min, 42ºC/30 min, 85ºC/5 min, finished with 4ºC.

#### Quantification of gene expression via qPCR

The cDNA synthesized was amplified with specific primers following standard protocols [33]. For qPCR, 5 μL of cDNA was amplified with the volume of specific primers corresponding to the optimum concentration (working solution of 10 μM, forward and reverse; Table 2), 12.5 μL of 2X SYBR Green Supermix (BioRad), and molecular grade water up to 25 μL. The reactions for each primer were performed using a CFX96 equipment (BioRad). A standard curve based on the PCR product was used for each primer as previously described [34] and results in R^2^ ≅ 1; reaction efficiency of 90–100%; a slope of ≅ −3.3. For *S. mutans* genes, the relative expression was calculated via normalization by 16S rRNA gene [33]. For *C. albicans* gene expression, the ACT1 gene was the first gene chosen as a normalizer for relative quantification [35]; but its expression in dual- and single-species of *C. albicans* biofilm was very different. Thus, the RPP2B (structural ribosome constituent) gene was selected as a normalizer [35], but, it also did not work as normalizer in this study because of the highly distinct expression level between the two types of biofilms. Therefore, the expression of specific *C. albicans* genes was only standardized using the same amount of RNA for cDNA synthesis and cDNA for qPCR.

**Table 2.** *Primers* for RT-qPCR.

Microorganism	Gene	Sequence of *Primers* (*forward* e *reverse*)	Tm (ºC)	Primer concentration (nM)	Product size (bp)	References
*S. mutans*	16S rRNA	ACCAGAAAGGGACGGCTAAC TAGCCTTTTACTCCAGACTTTCCTG	58	200	122	[6]
	*gtfB*	AAACAACCGAAGCTGATAC CAATTTCTTTTACATTGGGAAG	58	250	90	
	*nox1*	GGACAAGAATCTGGTGTTGA CAATATCAGTCTCTACCTTAGGC	58		91	[36]
	*atpD*	GGCGACAAGTCTCAAAGAATTG AACCATCAGTTGACTCCATAGC	58		115	[33]
*C. albicans*	ACT1	ATTCGGTGAGTAATCCTA GTATAGTCCAGATAACAACA	55	350	167	[37]
	RPP2B	TGCTTACTTATTGTTAGTTCAAGGTGGTA CAACACCAACGGATTCCAATAAA	60		83	[35]
	BGL2	ATGGGTGATTTGGCTTTCAA CAGCTGGACCAAGGTTTTGT	62		163	[38]
	PHR1	GGTTTGGTTCTGGTTGATGG AGCAGCAGTTCCTGGACATT	62		156	
	PHR2	CTCCTCCATTTCCAGAACCA CGTCTGAATCAACCTTGTCG	62		146	
	SOD1	TTGAACAAGAATCCGAATCC AGCCAATGACACCACAAGCAG	60	400	396	[22]

### Quantification of viable microbial cell numbers, biomass and biochemical characteristics of the biofilm matrix

At 43 h, biofilms were removed and evaluated to determine the dry-weight (biomass), total protein content, quantification of exopolysaccharides in the matrix [water-soluble (WSP) and -alkali soluble, ASP)], and the viable counts (CFU) [27,29]. The biofilms were washed three times with sterile 0.89% NaCl and transferred to a glass tube containing 2 mL of 0.89% NaCl. The tubes with the discs were sonicated for 10 min in an ultrasonic bath and the discs were scraped with sterilized stainless-steel spatulas. The suspension of each biofilm was transferred to a new 15 mL falcon tube, and the glass tubes were washed with 3 mL of 0.89% NaCl, which were also transferred to the falcon tube corresponding to each sample, totalizing 5 mL of biofilm suspension. The suspension was homogenized with a probe (30 s/7 w) (Sonicator QSonica, Q125).

From the volume of 5 mL, 0.1 mL was used for serial dilution and plating (on blood agar plate), followed by incubation (37ºC, 5% CO_2_, 48 h) and CFU counting. The remaining volume (4.9 mL) was centrifuged (4000 rpm, 20 min, 4°C – Eppendorf, Centrifuge 5810R). The supernatant was collected and stored in another tube (15 mL falcon) and the pellet (cells and the water-insoluble components of the extracellular matrix) was washed with 2.6 mL MilliQ water (4000 rpm, 20 min, 4°C; Eppendorf, Centrifuge 5810R). After centrifugation, the supernatant was transferred to the corresponding 15 mL falcon tube (which already contained supernatant). An additional wash was performed with 2.5 mL of water, and the resulting supernatant was stored (10 mL). The supernatants were precipitated with three volumes of 99% ethanol (18 h, −20°C), followed by three washes with 75% ethanol; the resulting pellets were air-dried, resuspended with 1 mL of water and used for quantification of WSP. The pellet of each biofilm was resuspended in 1.55 mL of water, of which 0.5 mL was used to calculate the insoluble dry-weight, 0.05 mL for quantification of total proteins (Bradford method), and 1 mL was used for ASP assessment. The biofilm aliquots for ASP extraction were dried (Speed Vac Concentrator RVC 2–18 CD Plus, Christ), the resulting pellets were weighted and used to extract ASP using 1 N NaOH (0.3 mL of 1 N NaOH per 1 mg of biofilm dry weight) followed by incubation (2 h, 37°C), and centrifugation (12,000 rpm, 10 min, 4ºC; Eppendorf, Centrifuge 5430R). The resulting supernatants were saved for analysis and the pellets were subjected to two more extraction procedures. The three supernatants per sample were combined and the extracted ASP were precipitated with three volumes of 99% ethanol (18 h, −20°C), followed by three washes with 75% ethanol; the resulting pellets were air-dried, and resuspended with the same volume of 1 N NaOH used for ASP extraction per sample. The quantification of WSP and ASP was performed using phenol-sulfuric acid colorimetric assay with glucose as standard [39].

### Stress tolerance assays

To investigate the response of *S. mutans* and *C. albicans* to different stress conditions, 43 h-old dual- and single-species biofilms were incubated in 1 mM glycine buffer with neutral (pH 7.0), acid pH (1 mM glycine buffer, pH 2.5), hydrogen peroxide (H_2_O_2_ 0.2%), and chlorhexidine digluconate (CHX 0.12%) during different periods (0, 30, 60 and 90 min) [40–42]. After each specific stress challenge and exposure time, the biofilms were processed as described above for CFU count. A total of eight biofilm sets were performed in duplicate (per type of biofilm) in at least three experimental occasions.

### Statistical analyses

The data were analyzed to examine whether there were differences between the dual- and single-species biofilms using Prism 7 software (GraphPad Software, Inc.). The analyses were performed using descriptive and inferential statistic according to the distribution (Shapiro–Wilk test of normality) using 5% of significance. Parametric data were subjected to one-way and two-way ANOVA (for more than two variables with Tukey pos-test) and the unpaired data t-test with Welch correction (for two variables). The non-parametric data were evaluated by Kruskal–Wallis test (for more than two variables with Dunn and Sidak post-test) or Mann Whitney test (for two variables). Data from viable counts recovered during stress challenges assays were evaluated by two-way ANOVA using as factors biofilm type (single- and dual-species), and exposure time (0, 30, 60, and 90 min) in two types of biofilms, followed by either Tukey’s multiple comparison test or Sidak’s multiple comparison test. Furthermore, the descriptive and qualitative analysis of confocal microscopy images was performed.

## Results

### pH of the spent culture media

**Figure 1.**
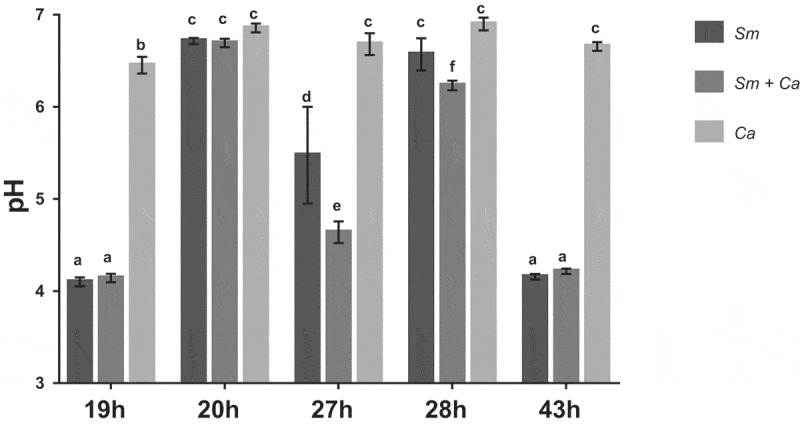
**pH of the spent culture media at distinct developmental phases of biofilms**. The data represent means and standard deviations (n = 12). Sm: *S. mutans*, Ca: *C. albicans*. Equal letters indicate statistically equal means by the Tukey test (p ≥ 0.05; two-way ANOVA, followed by Tukey test).

The pH of spent media was measured after the culture medium changes (19 and 27 h) and when the biofilm was removed for processing (19, 20, 28 and 43 h). There was a significant statistical difference between the three biofilms and developmental phases (p < 0.001; two-way ANOVA, followed by Tukey test). The mean pH values were more acidic at 19 and 43 h compared to 27 h for *S. mutans* single-species and dual-species biofilm (p < 0.001). *S. mutans* single-species and dual-species biofilm showed similar behavior at 19 and 43 h (p = 0.730 and p = 0.665). The pH values remained close to neutral at all phases for single-species *C. albicans* biofilms (p < 0.001) (Figure 1).

### 3D structure of single- and dual-species biofilms

**Figure 2.**
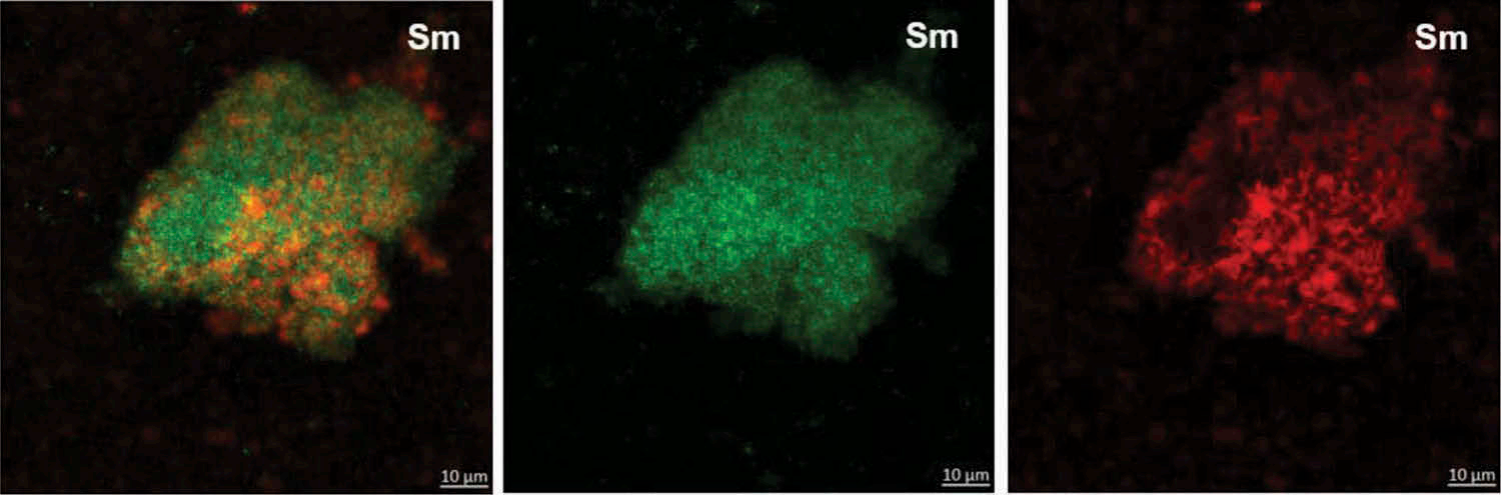
**Representative confocal microscopy images of 19 h-old *S. mutans* single-species biofilm**. The green color represents the *S. mutans* cells (GFP), while the red color represents exopolysaccharides in the extracellular matrix labeled with Alexa Fluor 647. In the first column overlay is observed, while the second and third illustrate each component individually.

**Figure 3.**
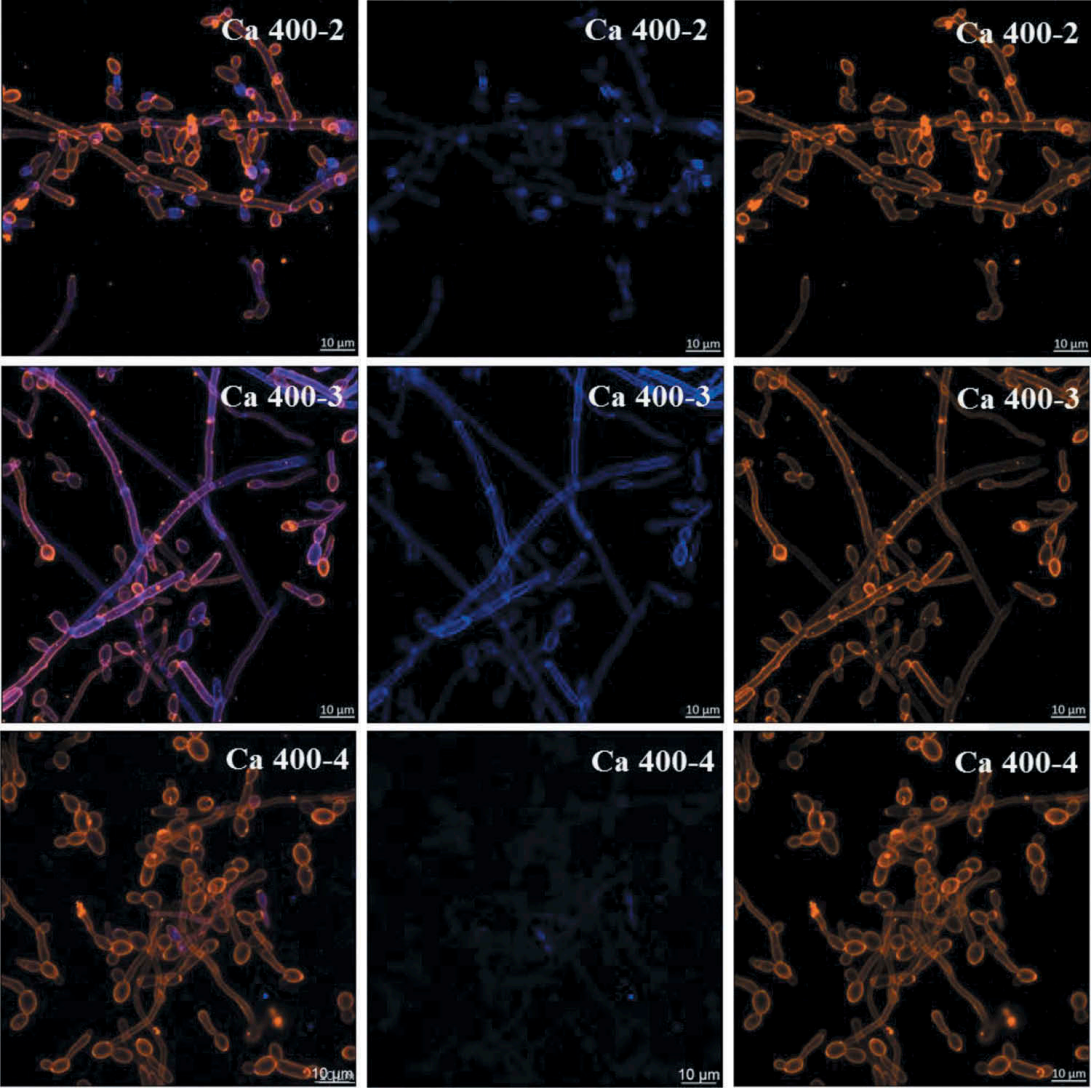
**Representative confocal microscopy images of 19 h-old *C. albicans* single-species biofilms**. These biofilms are labeled with primary antibody (400–2, 400–3, 400–4) paired with secondary antibody labeled with Alexa Fluor 405 (shown in blue). The orange color represents microbial cells of the biofilm labeled by lectin concanavalin A conjugated with TRITC. The blue color represents polysaccharides produced by *C. albicans*. In the first column the overlay is observed, while the second and third illustrate each component individually.

**Figure 4.**
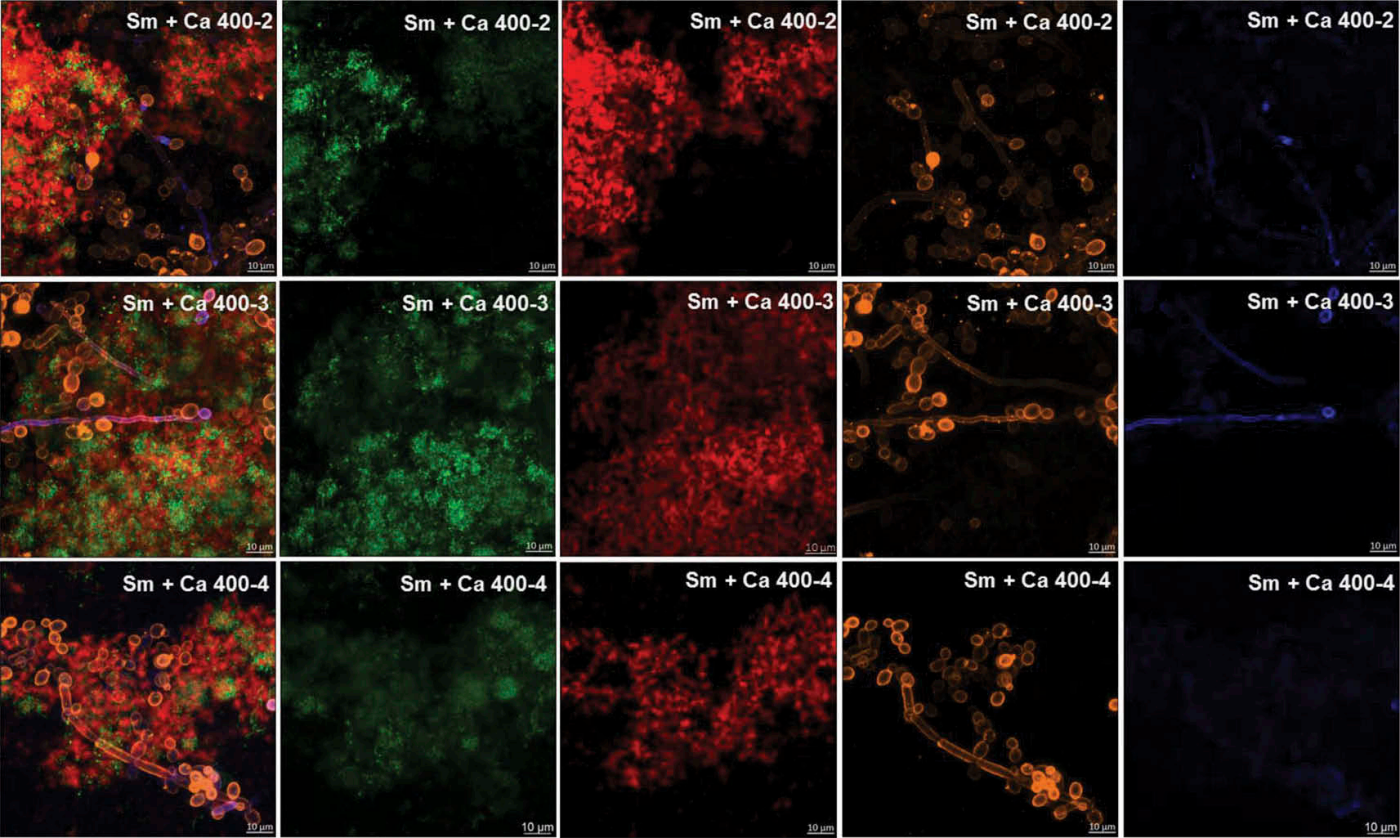
**Representative confocal microscopy images of 19 h-old *S. mutans* and *C. albicans* dual-species biofilms**. The green and orange colors represent cells of *S. mutans* (GFP) and *C. albicans* (concanavalin A conjugated with TRITC), respectively. The red color represents exopolysaccharides produced by *S. mutans* (Alexa Fluor 647) and the blue color represents polysaccharides produced by *C. albicans* (labeled with primary antibody 400–2, 400–3 or 400–4, and paired with secondary antibody conjugated to Alexa Fluor 405). The overlay is observed in the first column, while the other columns represent each component individually.

At 19 h, *S. mutans* single-species biofilm presented microcolonies (cluster of microbial cells) enmeshed and surrounded by and bacterium-derived exopolysaccharides (Figure 2). At this biofilm age, *C. albicans* single-species biofilms presented mostly hyphae cells that displayed higher labeling with antibodies for 1,3-β-glucans (400–2) and 1,3- and 1,4-β-glucan (400–3) (Figure 3), compared to lower intensity with antibody labeling 1,4-β-mannan and galacto-1,4-β-mannan (400–4). Controls demonstrated that there was no non-specific binding of the secondary antibody (Figure S1). Dual-species biofilms with the four markers displayed less labeled of polysaccharides produced by *C. albicans*, with higher intensity of label in hyphae cells (compared to oval cells – yeast) (Figure 4; Figure S2). It was also observed that microcolonies formed by *S. mutans* cells and *S. mutans*-derived exopolysaccharides matrix are located around *C. albicans* (wrapped mainly the hyphae morphology).

**Figure 5.**
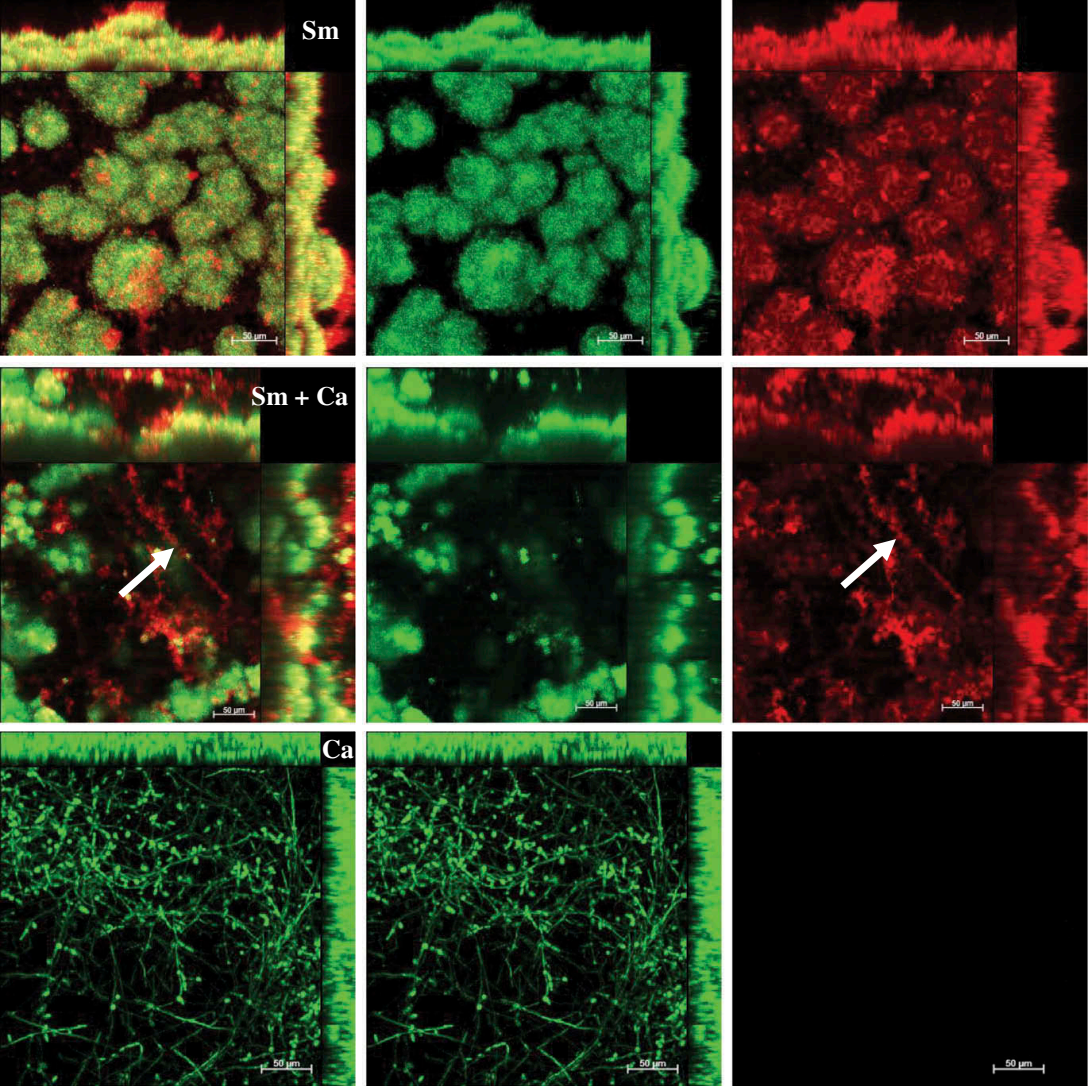
**Representative confocal microscopy images of 43 h-old *S. mutans* and *C. albicans* dual- and single-species biofilm**. The green color represents the microbial content in each biofilm (labeled with SYTO9), while the red color represents ESP in the extracellular matrix produced by *S. mutans* Gtfs (labeled with Alexa Fluor 647). The images in the first column show the overlay of both components, while the second and third images illustrate each component individually. The white arrow shows hyphae of *C. albicans* surrounded by exopolysaccharides produced by *S. mutans*. Sm: *S. mutans* and Ca: *C. albicans.*

At 43 h, the dual-species biofilm showed larger microcolonies than *S. mutans* single-species biofilm, whereas these structures were absent in *C. albicans* single-species biofilm (Figure 5). In dual-species biofilm, *C. albicans* cells were located around clusters of *S. mutans* cells. The distribution of exopolysaccharides was different in dual-species and *S. mutans* single-species biofilm, but as expected, this component cannot be visualized in biofilms of *C. albicans*. Dual-species biofilms were thicker than both single-species biofilms, as pointed out by the scale and orthogonal projections in Figure 5. In dual-species biofilms, *S. mutans-*derived exopolysaccharides are located around the hyphae (as indicated by white arrows in Figure 5).

### S. mutans and C. albicans gene expression in dual- and single-species biofilms

**Figure 6.**
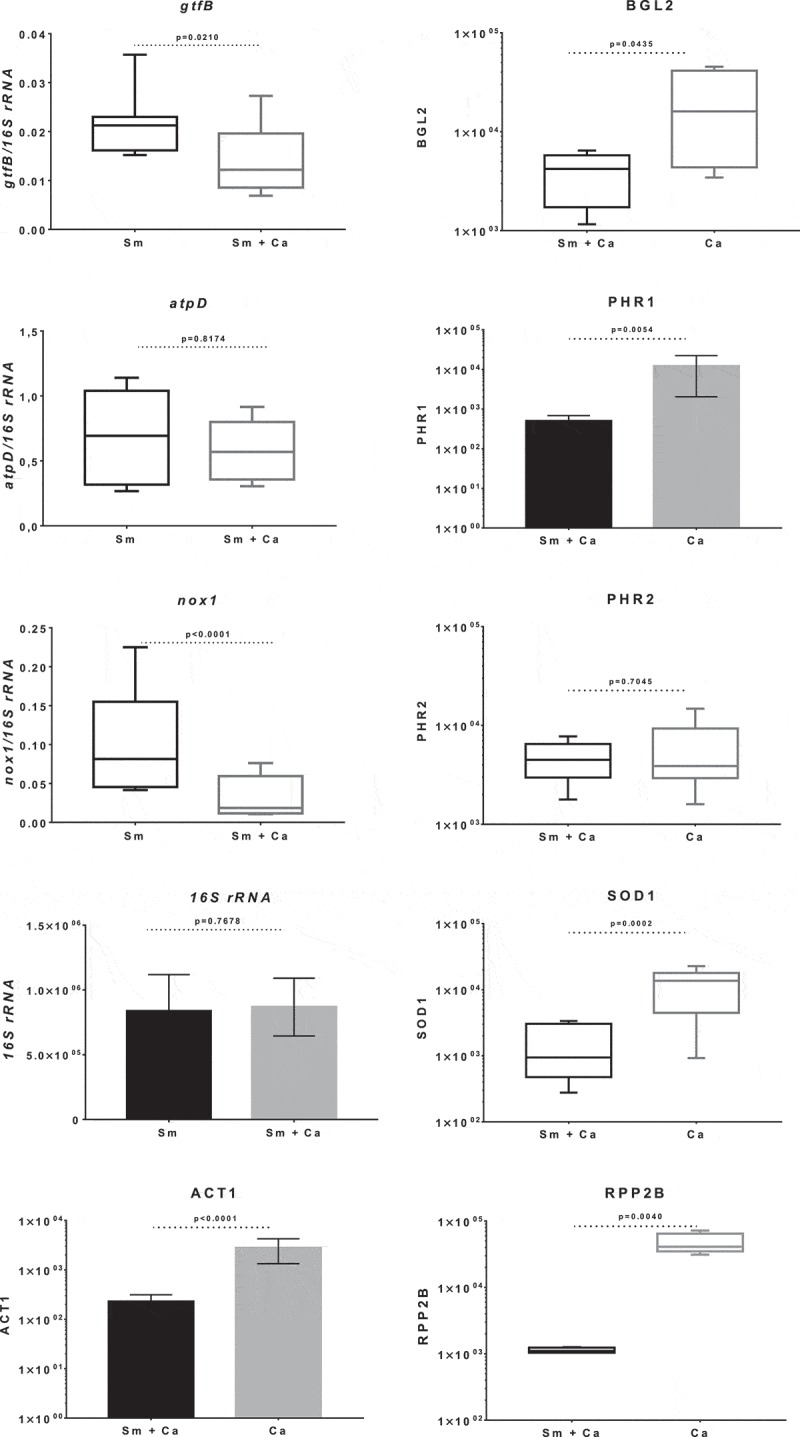
**Gene expression of *S. mutans* and *C. albicans* in 28 h-old single- and dual-species biofilms**. Data presented are mean and standard deviation for genes 16S rRNA, ACT1, PHR1 and RPP2B (bar graphs; unpaired data t-test with Welch correction), while median and interquartile range are shown for the other genes (box plot graphs, Mann Whitney test). The data were obtained from three experiments (with two cDNA per experiment), and the quantification of qPCR expression was performed in duplicate. Sm: *S. mutans* and Ca: *C. albicans.*

The expression of *S. mutans* (*gtfB, atpD*, and *nox1*, normalized by 16S rRNA) and *C. albicans* (BGL2, PHR1, PHR2, SOD1, ACT1, and RPP2B) genes is depicted in Figure 6. Genes of *C. albicans* were not normalized by ACT1 and RPP2B because there was a difference in the expression between dual-species and *C. albicans* single-species biofilm (as shown in Figure 6). Compared with dual-species biofilms, expression of *gtfB* and *nox1* was higher for *S. mutans* single-species while expression of BGL2, PHR1, and SOD1 was higher in *C. albicans* single-species. However, there was no difference between single- and dual-species biofilms for acid tolerance genes *atpD* (*S. mutans*) and PHR2 (*C. albicans*).

### Overall features of dual- and single-species biofilms

#### Viable counts of S. mutans and C. albicans

**Figure 7.**
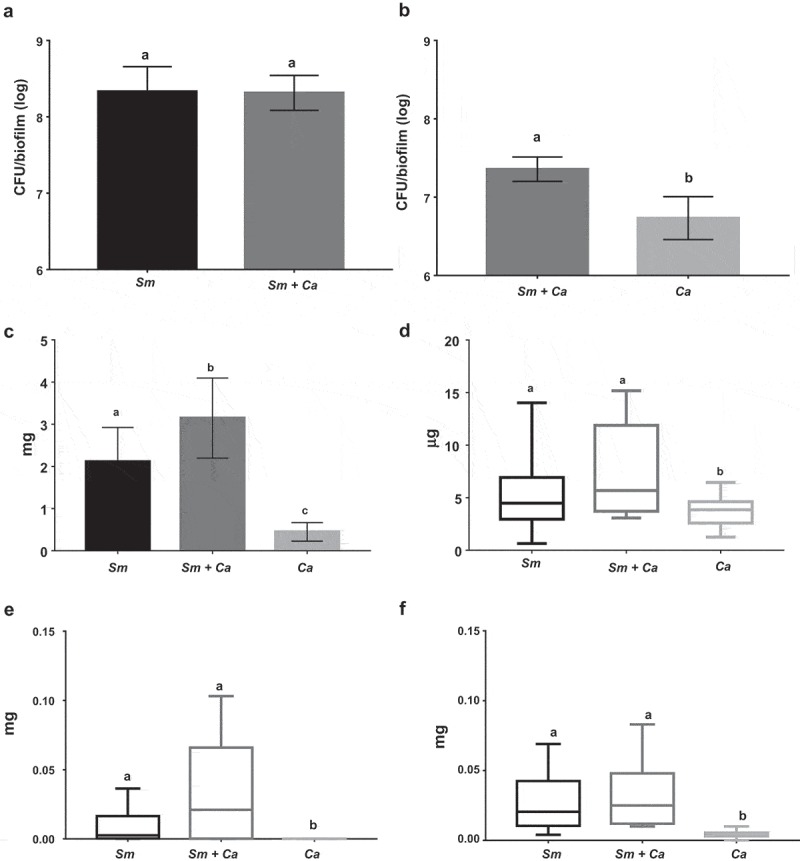
**Quantification of viable microbial cell numbers, biomass and exopolysaccharides in the matrix of 43 h-old *S. mutans* and *C. albicans* in single- and dual-species biofilms**. The *S. mutans* (A) and *C. albicans* (B) population are shown as average and standard deviation (n = 12). Graph C depicts the biofilms biomasses (insoluble dry-weight), as average and standard deviation (n = 12). Graph D shows protein data as median and interquartile range (n = 12). The exopolysaccharides in the matrix are water-soluble (E) and -insoluble (F) data as median and interquartile range. Similar letters indicate the absence of statistical difference per trait evaluated. Sm: *S. mutans* and Ca: *C. albicans.*

The quantification of viable microbial cell numbers of *S. mutans* and *C. albicans* are shown in Figure 7(a and b). There was no difference in the *S. mutans* counts in dual- and single-species biofilm (p = 0.831; unpaired t-test with Welch’s correction). However, there was a significant difference for *C. albicans* numbers, because the dual-species biofilm presented a higher amount of CFU/biofilm (log) when compared to the *C. albicans* single-species biofilm (p < 0.001, unpaired t-test with Welch’s correction).

#### Biomass (dry-weight insoluble) and proteins in biofilm

Dual-species biofilms show higher biomass, compared to *S. mutans* and to *C. albicans* single-species biofilms (a *vs*. b: p = 0.002; b *vs*. c: p < 0.001; one-way ANOVA, followed by Tukey test; Figure 7(c)). For proteins, dual-species biofilm presented elevated amounts compared to single-species biofilm, and this difference was statistically significant compared to *C. albicans* biofilm (a *vs*. b: p = 0.046; Kruskal–Wallis, followed by Dunn’s test; Figure 7(d)). However, less amount of proteins was observed in *C. albicans* biofilm.

#### Exopolysaccharides in the extracellular matrix

The amounts of WSP and ASP detected in the matrix of *S. mutans* and *C. albicans* dual- and single-species biofilms are depicted in Figure 7(e and f). There was no difference in the amount of WSP between *S. mutans* single-species and dual-species biofilms. However, *C. albicans* single-species biofilms presented less amount of WSP compared to the other two biofilm types (p < 0.001, Kruskal–Wallis, followed by Dunn’s test). The ASP amount was higher for dual-species biofilm compared to *C. albicans* single-species biofilm (p = 0.002). However, there was no difference in the amount of ASP between dual-species and *S. mutans* biofilms (p > 0.05; Kruskal–Wallis, followed by Dunn’s test).

### Stress tolerance

**Figure 8.**
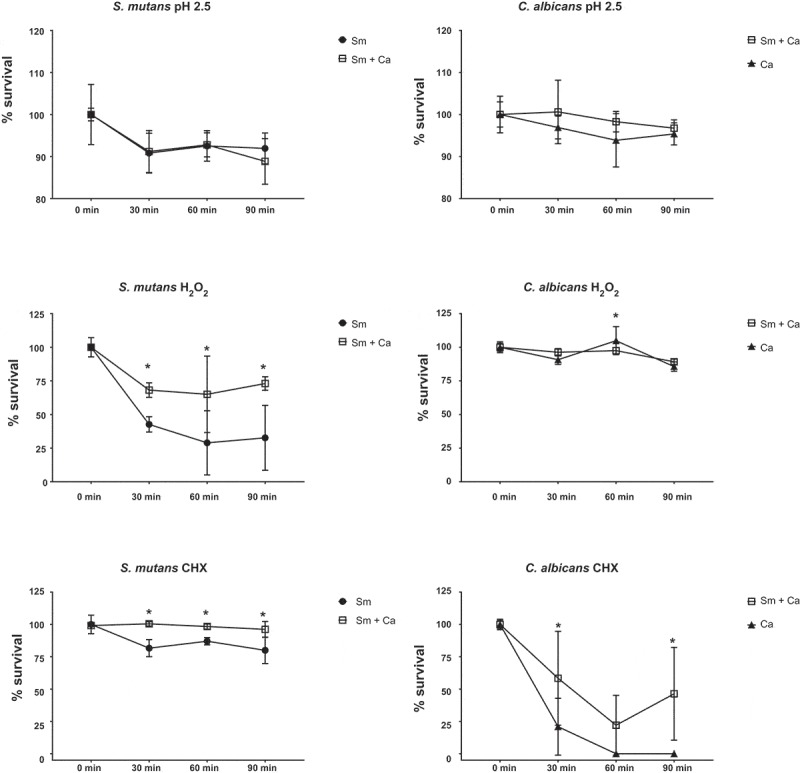
**Stress tolerance of *S. mutans* and *C. albicans* in dual- and single-species biofilms**. 43 h-old biofilms were longitudinally exposed (0, 30, 60 and 90 min) acid (pH 2.5), oxidative (0.2% H_2_O_2_) and antimicrobial (0.12% CHX) stresses. Data represent average and standard deviation of CFU/mL in percentage. *denotes statistical difference between a microbial species survival in dual- and single-species biofilms at a specific exposure time (two-way ANOVA, followed by Sidak’s multiple comparison tests). Statistical differences for the same biofilm type and per microorganism over time are not shown in the graphs but described in the main text. Sm: *S. mutans* and Ca: *C. albicans.*

Stress tolerance data are represented in Figure 8. Data from acid challenge demonstrated similar behavior in dual- and single-species biofilms for the two species (p > 0.05; two-way ANOVA). However, longitudinal analyses demonstrated that there were statistical differences for survival of *S. mutans* in dual- and single-species biofilms at 0 *vs*. 30, 60 and 90 (p ≤ 0.020; two-way ANOVA, followed by Tukey test) and *C. albicans* in single-species biofilms at 0 vs 60 min (p = 0.028; two-way ANOVA, followed by Tukey’s test).

Oxidative stress (H_2_O_2_) challenge lead to the statistical difference for *S. mutans* survival in dual- versus single-species biofilm (p ≤ 0.002; two-way ANOVA, followed by Sidak’s test). The bacterial survival over time in both biofilm types was decreased (0 min *vs*. 30, 60 and 90 min) (p ≤ 0.019; two-way ANOVA, followed by Tukey’s test). Interestingly, *C. albicans* presented similar tolerance to H_2_O_2_ exposure in dual- and single-species biofilms (p > 0.05), except at 60 min (p = 0.001). Nevertheless, *C. albicans* counts in single-species biofilm presented statistical difference between 0 min *vs*. 30 and 90 min; and between 60 min *vs*. 30 and 90 min (p ≤ 0.0004; two-way ANOVA, followed by Tukey test); while counts in dual-species biofilm displayed statistical difference between 0 *vs*. 30 min, 30 and 60 *vs*. 90 min (p ≤ 0.003; two-way ANOVA, followed by Tukey test).

In addition, the antimicrobial challenge data showed a statistical difference between biofilm types (dual- *vs* single-species) for *S. mutans* and *C. albicans* at 30, 60 and 90 min (p ≤ 0.004; two-way ANOVA, followed by Sidak’s test), except at 60 min for *C. albicans* (p > 0.05). Specifically, there was a decrease in bacterial survival over the time in single-species biofilms (0 min *vs*. 30, 60 and 90 min, p < 0.0001; two-way ANOVA, followed by Tukey’s test), while there was no difference for *S. mutans* survival in dual-species biofilms (p > 0.05). The fungus in single-species biofilms presented a steep decrease in survival from 0 to the other exposure times (p < 0.0001; two-way ANOVA, followed by Tukey’s test), while in dual-species its survival also decreased over time, but not as pronounced (p ≤ 0.006; two-way ANOVA, followed by Tukey’s test).

## Discussion

Understanding the interaction of microorganisms in pathogenic biofilms is paramount to develop prevention and control strategies. Thus, the present study characterized *in vitro* single- and dual-species of *S. mutans* and *C. albicans*. The data demonstrated a similar behavior for pH of spent medium of *S. mutans* single-species and the dual-species biofilms. However, other parameters such as viable counts of microbial cells, exopolysaccharides (essential for biofilm formation), biomass, proteins, 3D structure and stress tolerance (mainly to oxidative and antimicrobial challenges) revealed greater complexity for dual-species biofilms, corroborating previous studies [14,15,26] but not the research that hypothesized that *C. albicans* could prevent caries [43]. This hypothesis was suggested because *C. albicans* produces ethanol when metabolizing sucrose, which does not influence on pH of the medium. Further, in periods of scarcity and/or hypoxia this fungus is forced to use lactic acid as a source of carbon (energy), corroborating with the elimination of acids in *S. mutans* and *C. albicans* dual-species biofilms and consequent reduction of dental demineralization [43]. This discrepancy may be due to the *in vitro* models used in each study.

In the current study, the same microbial load per species was used to form single- and dual-species biofilms. Thus, the total biomass (total microbial load) was higher to initiate dual-species biofilms. Nevertheless, the biofilm model used is based on active microbial adhesion on hydroxyapatite surface because the discs were placed vertically suspended inside the wells of 24-well plates. Therefore, in the dual-species biofilms *S. mutans* and *C. albicans* had the same surface area available that each species had in the single-species setting for adhesion and consequent biofilm development. The pH of the culture medium was more acidic in dual-species biofilms at 27 h, 10 h after the medium change; but, pH data at 19 and 43 h that reflect higher incubation time (19 h for 19 h and 14 h for 43 h) are similar for *S. mutans* single-species and dual-species biofilms. In the dual-species biofilm, the metabolism of both microorganisms leads to lower pH, mainly via metabolism of sucrose by *S. mutans* and glucose by *C. albicans*. The fungus *C. albicans* does not ferment well sucrose, and therefore the pH in the single-species biofilm remained neutral at all developmental phases under the tested experimental conditions. Thus, in the dual-species biofilm, the acid pH may be a result of the metabolism of sucrose by *S. mutans* and the released glucose and fructose is then available for *C. albicans*. Moreover, in acidic environments *C. albicans* produces proteinases that can destroy collagen in dentin caries and facilitate the fungi invasion inside dentinal tubules [44]; thus, explaining *in vivo* caries lesions in humans and rodents [14,15,26].

The extracellular matrix is critical for the formation of the biofilm and its virulence because it limits the diffusion of substances from inside to outside of the biofilm and the opposite, so the acid, responsible for the dissolution of the enamel, accumulates, and the saliva cannot properly exercise its function of neutralization. *S. mutans* can easily form acidic microenvironments in biofilm, being the main exopolysaccharides producer for the matrix [45]. There was a higher amount of *S. mutans*-derived exopolysaccharides in the matrix of dual-species biofilms (Figure 5, orthogonal projections), corroborating with previous studies [14,15,26]. *S. mutans* secretes Gtfs enzymes that synthesize WPS and ASP (glucans) [4]. Gtfs can be components of the salivary pellicle and adsorbed on the surfaces of other oral microorganisms. These enzymes synthesize glucans on the dental surface and the microbial surfaces improving the adhesion and accumulation of microorganisms on the teeth. *C. albicans* is one of the microorganisms to which Gtfs bind, particularly GtfB, and form significant amounts of exopolysaccharides in the presence of sucrose, which favors the adhesion and colonization of other microorganisms [16]. Furthermore, polysaccharides produced by *C. albicans* (1,3-β-glucans; 1,4-β-glucans; 1,4-β-mannan and galacto 1,4-β mannan) were observed in 19 h-old biofilm (Figures 3 and 4), although the contribution of this fungus to exopolysaccharides in the extracellular matrix may be to a lesser extent than *S. mutans*, as shown before for 1,3-β-glucans in older biofilms [14].

Here, *C. albicans* single-species biofilm presented smaller structure and greater dispersion of its cells on hydroxyapatite surface, compared to dual-species and *S. mutans* single-species biofilms. However, *C. albicans* appears to produce more polysaccharides (mostly 1,3- and 1,4-β glucans) when grown as single-species (Figure 3 *vs*. Figure 4). *C. albicans* single-species biofilm presented hyphae as the predominant cell morphology. Thus, the higher intensity of labeling in hyphae compared to yeast cells could be because the composition of exposed cell wall structures may differ in the distinct fungal cell morphologies [46]. Besides, *C. albicans* polysaccharides labeled with specific antibodies was lower in the dual-species biofilms. This behavior may be because (i) the antibody access to these targets was being restricted in the dual-species biofilms; (ii) there may be a link between 1,3-β- and 1,4- β-glucans of *C. albicans* and *S. mutans*-derived exopolysaccharides, preventing labeling with the antibodies studied; and/or (iii) *C. albicans* benefits when in association with bacteria (commensalism), decreasing the production of its polysaccharides (confirmed by gene expression data of BGL2).

Moreover, older biofilms were also subjected to biochemical assays to quantify exopolysaccharides. Although the amount of ASP detected by biochemical reaction was higher for dual-species biofilm it was not statistically different from *S. mutans* single-species biofilms (Figure 7F). The apparent contradiction in the amount quantified by biochemical assay and the confocal images can be because of the distribution of exopolysaccharides interspaced between *S. mutans* cells (microcolonies) and *C. albicans* cells. Furthermore, the work by Falsetta et al. [14] quantified *S. mutans-*derived exopolysaccharides via confocal analysis in single- and dual-species but did not use biochemical assay (phenol and sulfuric acid assay [39];). Additional studies have used the referred biochemical assay [15,26]. Hwang et al. [15] quantified the total water-insoluble exopolysaccharides in 18 and 42 h-old dual-species biofilms formed with distinct *C. albicans* mutant strains but did not report the amount in *S. mutans* single-species biofilms. In addition, Kim et al. [26], evaluated these polysaccharides only from dual-species biofilms subjected to distinct topical treatments. Thus, the data presented here is original for exopolysaccharides (ASP and WSP), based on biochemical quantification for single- versus dual-species biofilms.

Differences in gene expression can demonstrate how *S. mutans* handles and thrives in an acidified environment in the presence of *C. albicans*, and vice-versa. Initially, biofilms were processed for RNA isolation at 20 and 28 h (1 h after the culture media change at 19 or 27 h), to obtain similar pH of spent media to avoid media pH influence in the gene expression profile. However, the yield of *C. albicans* single-species biofilms was meager at 20 h, even after combining biofilms from 15 discs (data not shown). Therefore, the experiments were performed with 28 h-old biofilms. Previous studies were conducted for *S. mutans* genes by comparing *S. mutans* single-species and *S. mutans* and *C. albicans* dual-species biofilms at distinct developmental phases [14,26,47]. However, the expression of *C. albicans* genes in *S. mutans* and *C. albicans* dual-species biofilms has not been reported yet.

The presence of *C. albicans* in biofilms has been shown to induce the expression of *S. mutans* Gtfs, increasing the colonization of microorganisms, the amount of *S. mutans*-derived exopolysaccharides in the biofilm matrix and the biofilm virulence [1,14,48]. These factors may justify the higher amount of exopolysaccharides, biomass, proteins, and viable microbial cell numbers seen in dual-species biofilms. However, the expression level of *S. mutans* genes *gtfB* and *atpD* in 28 h-old dual-species biofilms was different from that found previously in biofilms with other ages (22, 32 e 42 h) [14,26,47]. Specifically, in these previous studies the level of both *gtfB* and *atpD* genes was higher in dual-species biofilms versus *S. mutans* single-species biofilms, but here *gtfB* level was higher in single-species while *atpD* was similar in both biofilms. A possible explanation for this difference is that the biofilm age was different. In addition, the expression of *nox1* was also higher here in *S. mutans* single-species biofilms. The gene products of *atpD* and *nox1* are involved in metabolic pathways for acid and oxidative stresses tolerance [33], and their expression may affect how *S. mutans* cope with stresses in single- and dual-species biofilms.

For *C. albicans*, the expression of genes that could contribute to the formation of virulent biofilms in the context of kingdom interactions (bacterium-fungus) was also evaluated. These genes included the glucan transferases BGL2 (synthesis of 1,3-β-glucans, cell wall biogenesis) [19], the glycosidase PHR1 (that may act on cell-wall beta-1,3-glucan prior to beta-1,6-glucan linkage and stress tolerance when pH ≥ 5.5) [19,21]. In addition, the glycosidase PHR2 (associated with acid stress tolerance and induced at pH ≤ 5) [20,21], and SOD1 (associated with oxidative stress tolerance) [19,20]. There was no difference in the expression of gene PHR2 in single- and dual-species biofilms. However, high levels of gene expression were found in *C. albicans* single-species biofilms for BGL2 and PHR1, corroborating with confocal images that show increased labeling of polysaccharides when *C. albicans* was alone (*vs*. dual-species biofilms). In addition, SOD1 and two putative genes described as normalizers ACT1 and RPP2B [35] were more abundantly expressed in single-species biofilms. As there was a greater development in the structure of dual-species biofilms (confirmed by confocal microscopy, viable counts of microbial cells, extracellular matrix composition and justified by the stress challenges), the analysis of gene expression indicates that there may be a ‘delay’ in the metabolism of the single-species biofilms of both species at the biofilm age evaluated. Thus, the expression profile of *C. albicans* genes in single-species biofilms compared to dual-species biofilms is quite distinct and further studies are needed to better understand the factors driving these differences, which could help to devise better control strategies.

In stress challenges, acid stress data of *S. mutans* and *C. albicans* corroborate with gene expression profile of the acid stress genes (*atpD* and PHR2 genes). *S. mutans* can adapt well to changes in pH mainly due to its ability to withstand acidic environments via increased activity of the F1F0-ATPase system (a primary mechanism to maintaining intracellular homeostasis), the capacity to repair DNA at low pH among other mechanisms of tolerance [49,50]. The expression of genes linked to oxidative stress responses (*nox1* and SOD1) also confirm the results of the challenge to oxidative stress (H_2_O_2_) for *S. mutans*, but not for *C. albicans*. This finding could be because the fungus presents an arsenal of genes linked to oxidative stress tolerance [51]. The more susceptible behavior of *S. mutans* in single-species biofilms under conditions of oxidative stress has been reported previously [41,52]. Reduced cell viability of *S. mutans* and at some extent *C. albicans* in single-species biofilms after oxidative challenge may be also related to the biofilms’ 3D architecture. Furthermore, both bacterium and fungus are more susceptible to killing by CHX in single-species biofilms, which may be linked to the lower amount of negatively charged exopolysaccharides for sequestering the cationic drug [6]. Therefore, strategies with targets for oxidative stress tolerance pathways and agents that can be trapped by the extracellular matrix charge may not be effective against dual-species biofilms of *S. mutans* and *C. albicans.*

In summary, *S. mutans* and *C. albicans* dual-species biofilms are more complex, structured and exhibit organized extracellular matrix that makes both species more tolerant to environmental stresses. Thus, the interaction of *S. mutans* and *C. albicans* can benefit both species in biofilms. Future studies should look for possible new therapeutic targets against this type of biofilm.
