# Supplementary material for: Dual-species biofilms of Streptococcus mutans and Candida albicans exhibit more biomass and are mutually beneficial compared with single-species biofilms
Source: J Oral Microbiol. 2019 Mar 20;11(1):1581520. doi: 10.1080/20002297.2019.1581520 (PMC6807867; doi:10.1080/20002297.2019.1581520)
Supplement: Supplemental Material [file ZJOM_A_1581520_SM3675.docx]

**Supplemental Material**


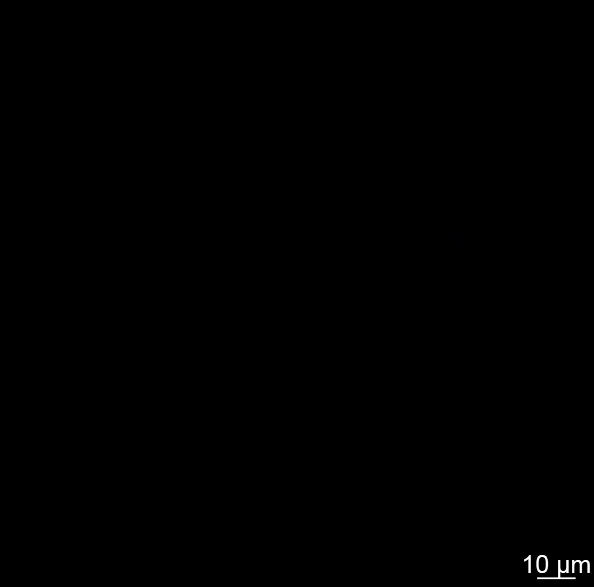

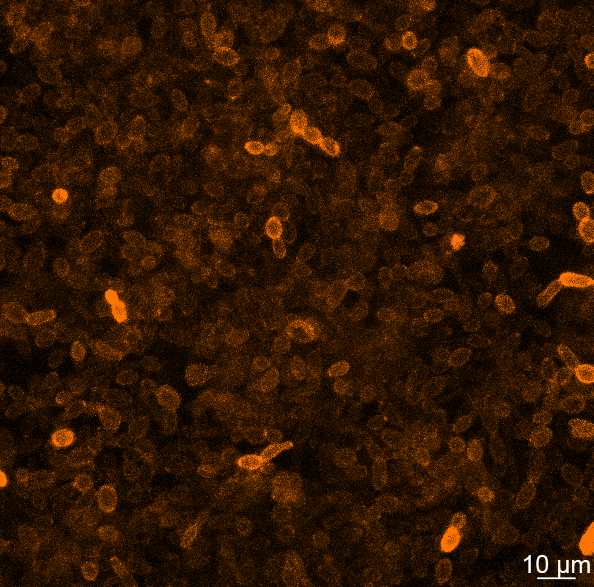

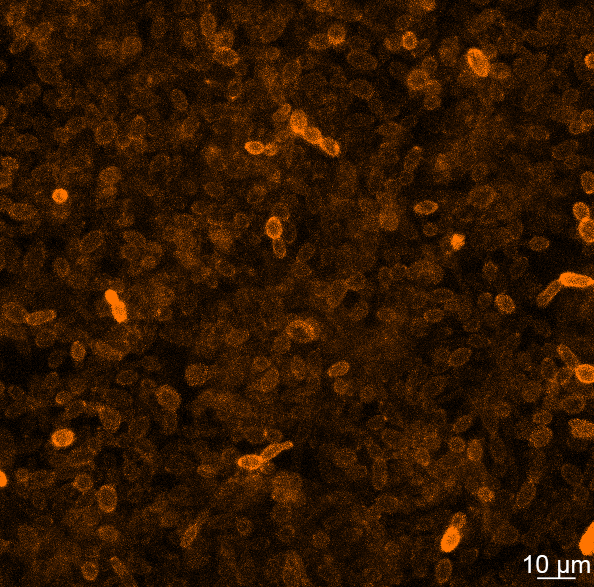

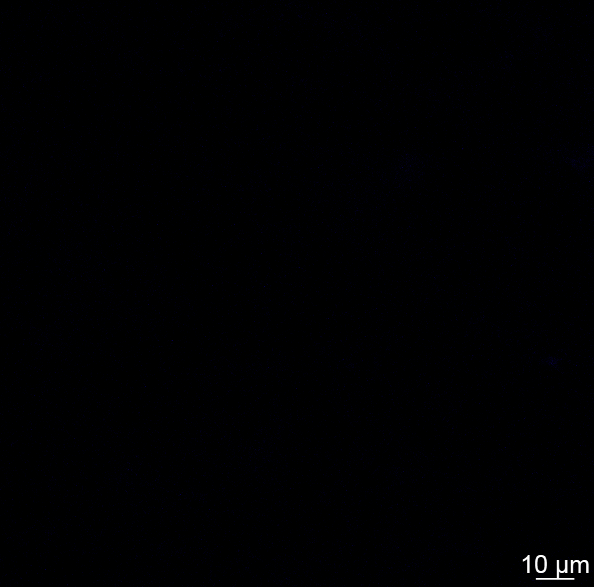


**Ca 400-2**


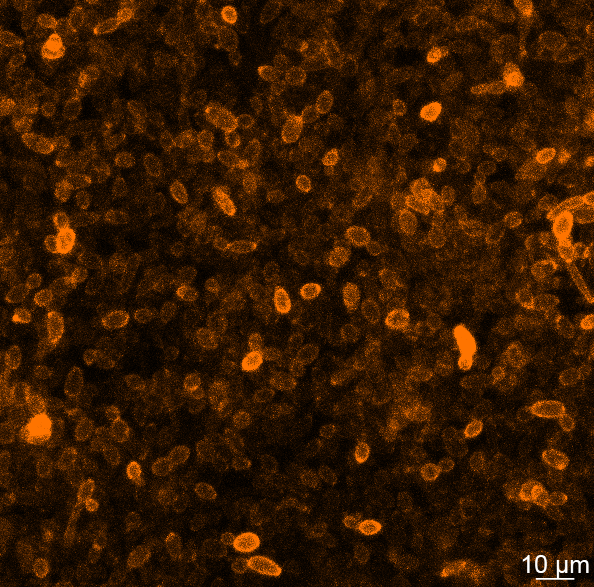


**Ca 400-2**


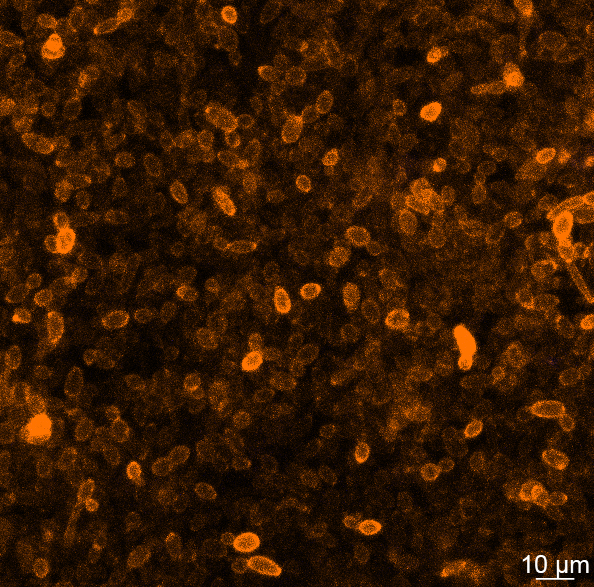


**Ca 400-2**


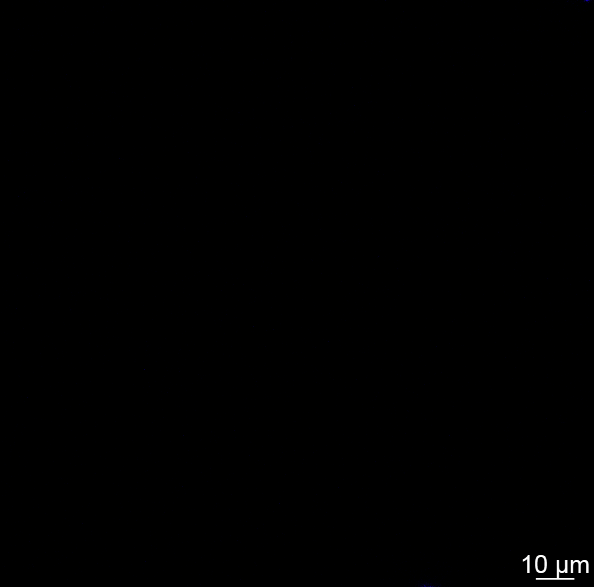


**Ca 400-3**


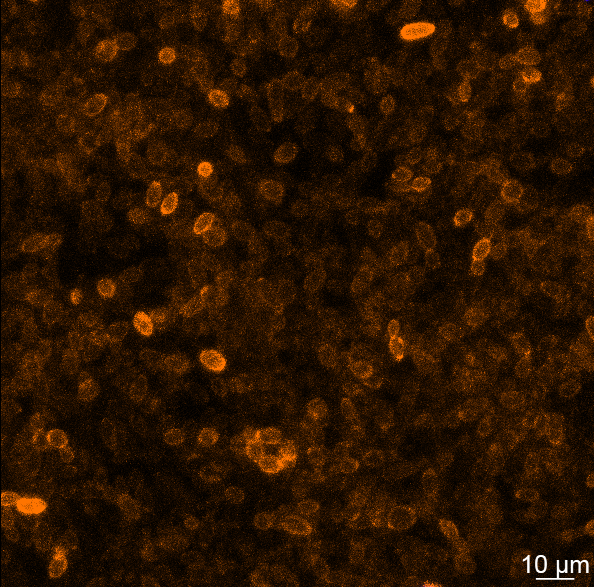


**Ca 400-3**


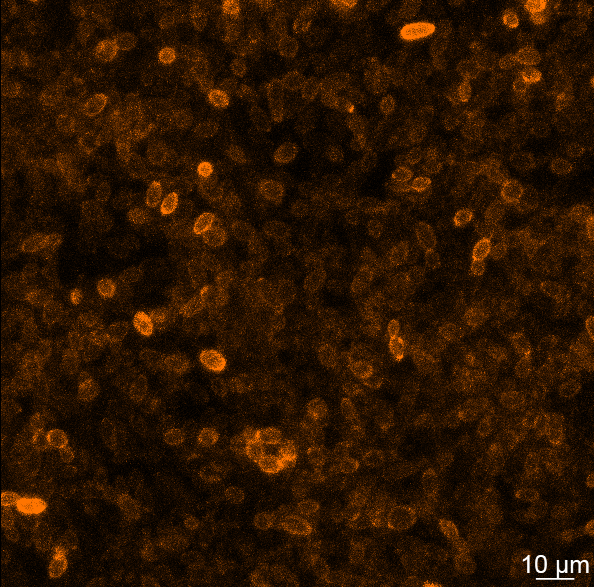


**Ca 400-3**

**Ca 400-4**


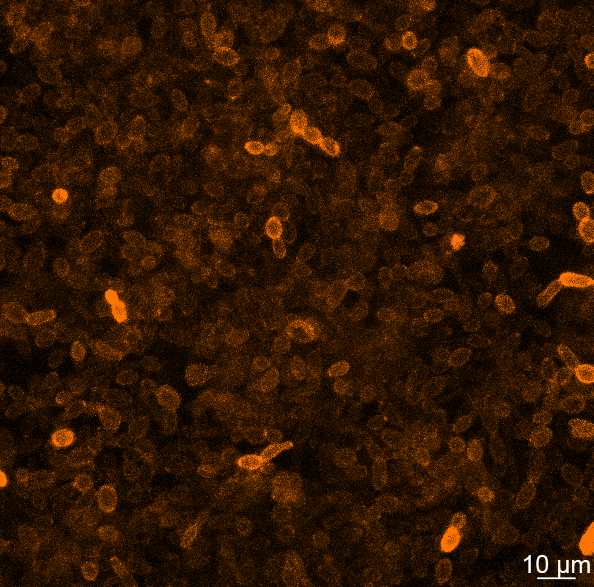


**Ca 400-4**

**Ca 400-4**

**Figure S1. Representative confocal microscopy images of *C. albicans* single-species biofilms as controls for fungal polysaccharides labelling.** The orange color represents cells of *C. albicans* labelled with concanavalin A conjugated with TRITC. The blue color should represent polysaccharides produced by *C. albicans* (labelled with primary antibody 400-2, 400-3 or 400-4, and paired with secondary antibody conjugated to Alexa Fluor 405). Here, the blue color is absent, which demonstrate that in the absence of the correct substrate, no fluorescence was detected.


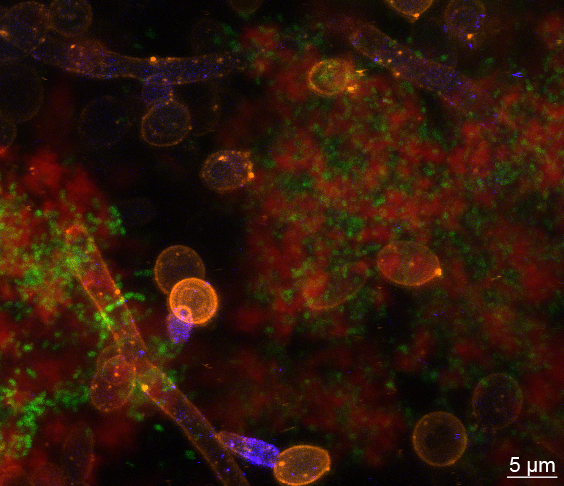

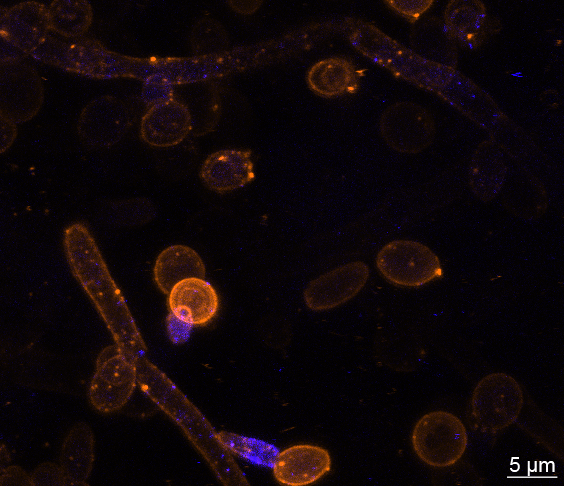

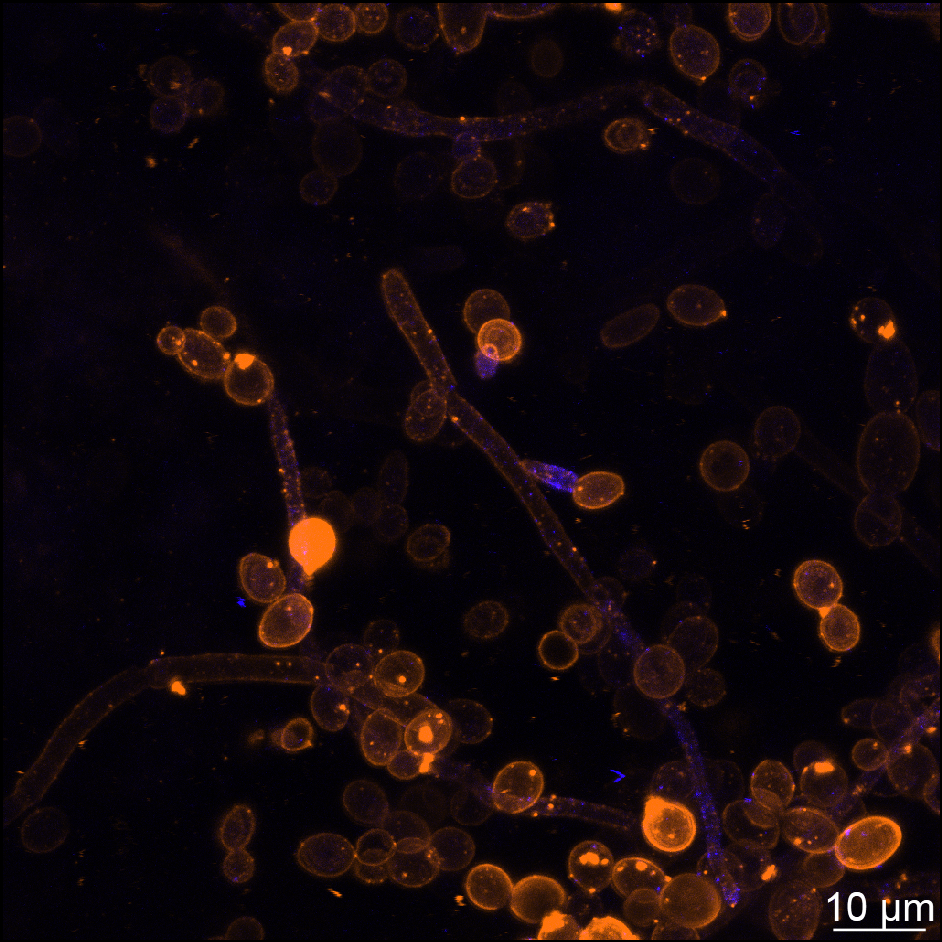


**Sm + Ca 400-2**


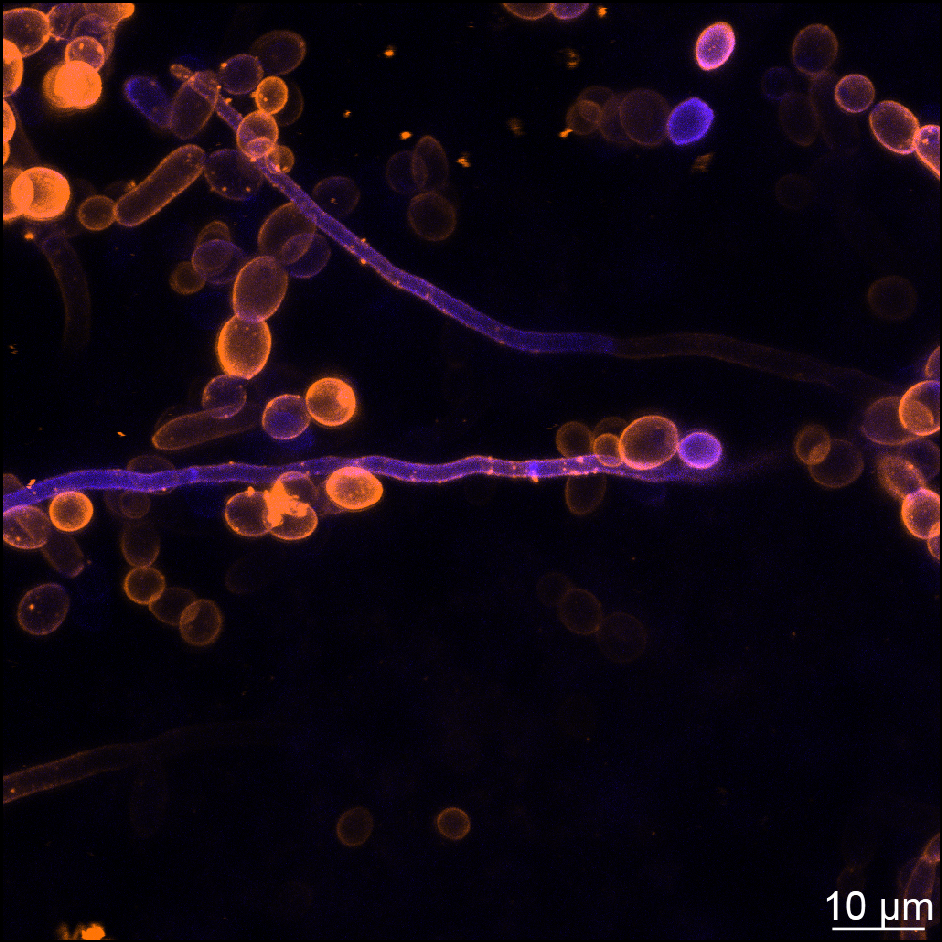


**Sm + Ca 400-3**


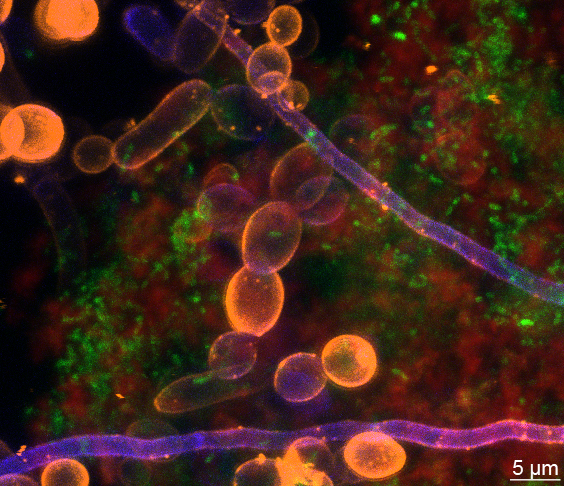

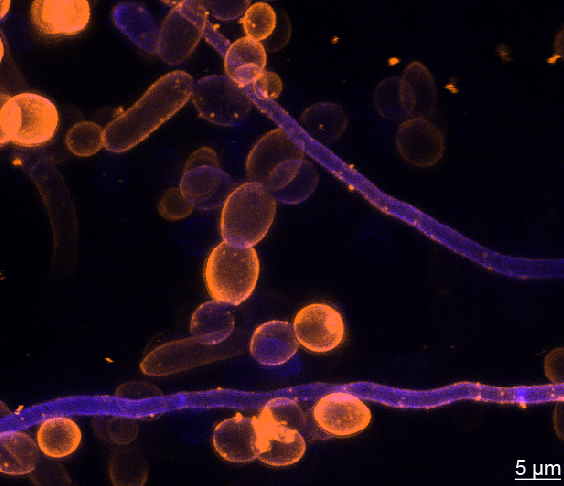

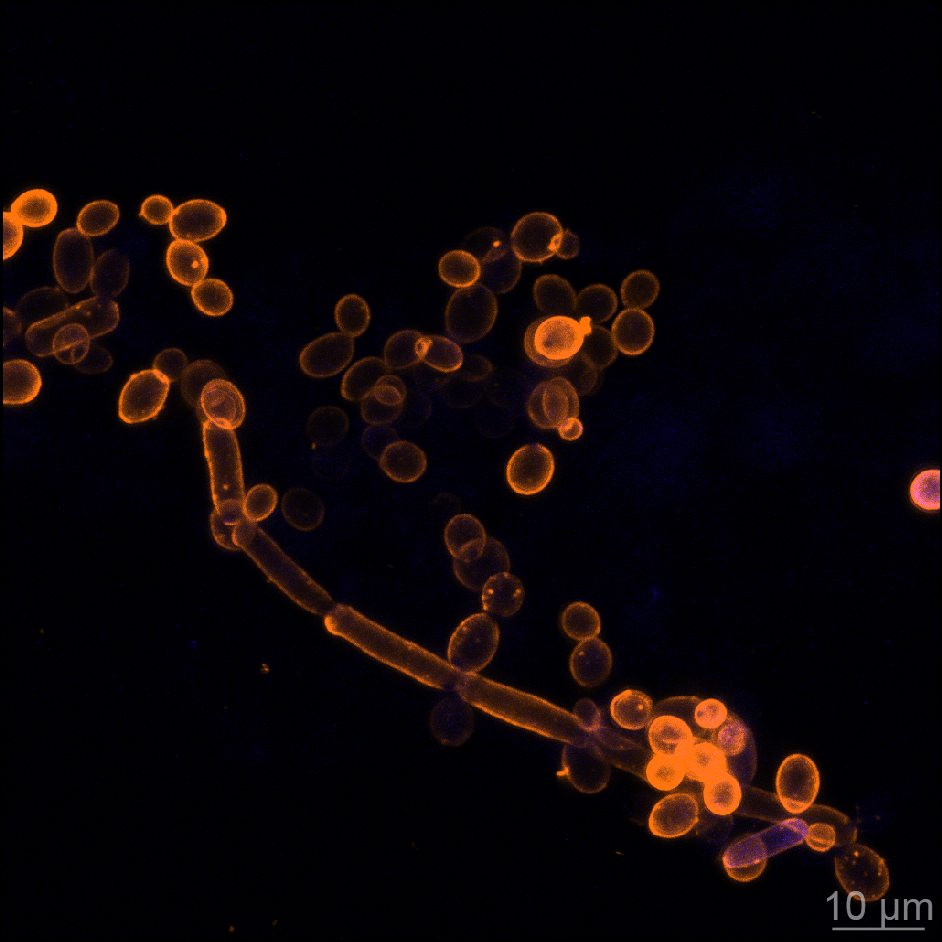


**Sm + Ca 400-4**


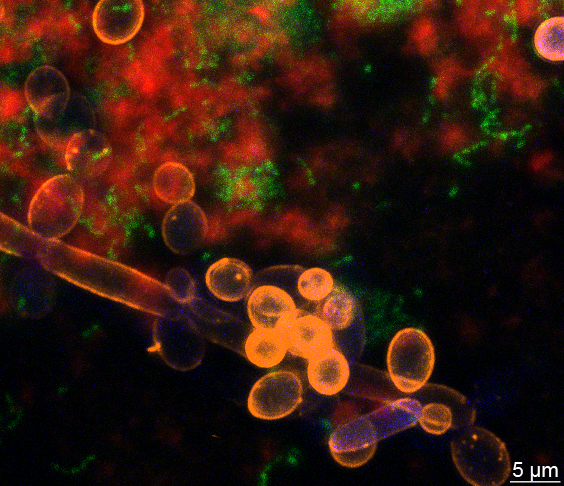

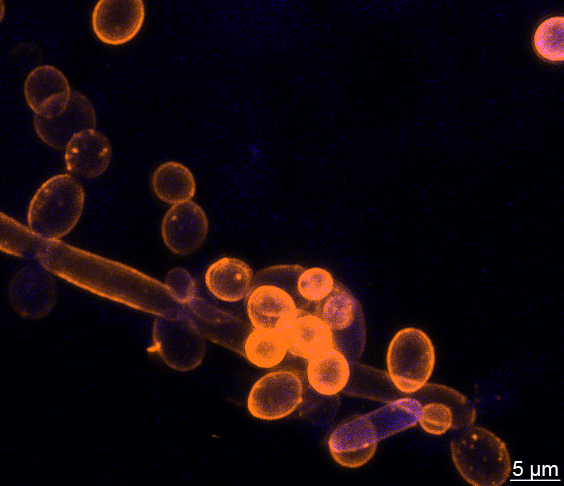


**Figure S2. Representative confocal microscopy images of 19 h-old *S. mutans* and *C. albicans* dual-species biofilms to highlight labelling of *C. albicans* structures.** The fist column depicts an overlay *C. albicans* cells (orange; labelled with concanavalin A conjugated with TRITC) and polysaccharides produced by *C. albicans* (blue; labelled by primary antibodies 400-2, 400-3 or 400-4 paired with secondary antibody conjugated to Alexa Fluor 405). The second column shows the cropped areas of each image to enhance the visualization of polysaccharides produced by *C. albicans.* The third column depicts overlay images of the four labelled structures. The green color represent cells of *S. mutans* (GFP). The red color represents exopolysaccharides produced by *S. mutans* (Alexa Fluor 647).
